# A pharmaco-metabolomics approach in a clinical trial of ALS: Identification of predictive markers of progression

**DOI:** 10.1371/journal.pone.0198116

**Published:** 2018-06-05

**Authors:** Hélène Blasco, Franck Patin, Amandine Descat, Guillaume Garçon, Philippe Corcia, Patrick Gelé, Timothée Lenglet, Peter Bede, Vincent Meininger, David Devos, Jean François Gossens, Pierre-François Pradat

**Affiliations:** 1 Université François-Rabelais, Inserm, Tours, France; 2 Laboratoire de Biochimie, CHRU de Tours, Tours, France; 3 Centre Universitaire de Mesures et d'Analyses (CUMA), EA, Université de Lille, Lille, France; 4 Université de Lille, CHU Lille, Institut Pasteur de Lille, EA, IMPECS, Lille, France; 5 Centre SLA, Service de Neurologie, CHRU Bretonneau, Tours, France; 6 Centre d'Investigation Clinique, Université de Lille, Lille, France; 7 Département des Maladies du Système Nerveux, Centre Référent Maladie Rare SLA, Hôpital de la Pitié-Salpétrière, Paris, France; 8 Sorbonne Universités, UPMC Univ Paris 06, CNRS, INSERM, Laboratoire d’Imagerie Biomédicale,Paris, France; 9 Academic Unit of Neurology, Trinity College, Dublin, Ireland; 10 Ramsay, Hôpital des Peupliers, Paris, France; 11 INSERM U1171, Pharmacologie Médicale & Neurologie, Université, Faculté de Médecine, CHU de Lille, Lille, France; 12 Northern Ireland Centre for Stratified Medicine, Biomedical Sciences Research Institute Ulster University, C-TRIC, Altnagelvin Hospital, Derry/Londonderry, United Kingdom; Macquarie University, AUSTRALIA

## Abstract

There is an urgent and unmet need for accurate biomarkers in Amyotrophic Lateral Sclerosis. A pharmaco-metabolomics study was conducted using plasma samples from the TRO19622 (olesoxime) trial to assess the link between early metabolomic profiles and clinical outcomes. Patients included in this trial were randomized into either Group O receiving olesoxime (n = 38) or Group P receiving placebo (n = 36). The metabolomic profile was assessed at time-point one (V1) and 12 months (V12) after the initiation of the treatment. High performance liquid chromatography coupled with tandem mass spectrometry was used to quantify 188 metabolites (Biocrates® commercial kit). Multivariate analysis based on machine learning approaches (i.e. Biosigner algorithm) was performed. Metabolomic profiles at V1 and V12 and changes in metabolomic profiles between V1 and V12 accurately discriminated between Groups O and P (p<5×10–6), and identified glycine, kynurenine and citrulline/arginine as the best predictors of group membership. Changes in metabolomic profiles were closely linked to clinical progression, and correlated with glutamine levels in Group P and amino acids, lipids and spermidine levels in Group O. Multivariate models accurately predicted disease progression and highlighted the discriminant role of sphingomyelins (SM C22:3, SM C24:1, SM OH C22:2, SM C16:1). To predict SVC from SM C24:1 in group O and SVC from SM OH C22:2 and SM C16:1 in group P+O, we noted a median sensitivity between 67% and 100%, a specificity between 66.7 and 71.4%, a positive predictive value between 66 and 75% and a negative predictive value between 70% and 100% in the test sets. This proof-of-concept study demonstrates that the metabolomics has a role in evaluating the biological effect of an investigational drug and may be a candidate biomarker as a secondary outcome measure in clinical trials.

## Introduction

Amyotrophic Lateral Sclerosis (ALS) is the most common motor neuron disorder and the commonest neurodegenerative condition in young adults. Disease heterogeneity, lack of validated biomarkers and limited understanding of etiological factors hamper drug development efforts. Current clinical trials of ALS overwhelmingly rely on functional scales and survival and clinical trial design shortcomings may have contributed to limited progress in drug-development. [1]. The urgency of developing and validating sensitive biomarkers has been repeatedly emphasized, and there is a particular need for panels of markers which can detect subtle changes over relatively short periods of time. Among the plethora of wet and dry biomarkers which have been proposed in ALS, metabolomics has emerged as one of the most promising “omics” approach due to the evaluation of disease-specific metabolic signatures. Pharmaco-metabolomics is the study of metabolomic profile (metabotype) alterations associated with therapy and is a key conceptual approach in the era of precision medicine. Advances in metabolomic profiling have led to successful clinical trial applications and are now used as secondary outcome measures in some clinical trials [2, 3]. Given the close association between the metabolomic profile and disease stage, metabolomics is a strong candidate biomarker and robust surrogate endpoint for clinical trials.

Olesoxime is a small molecular weight chemical compound with neuroprotective and neurodegenerative properties [4, 5]. This cholesterol-like molecule is likely to affect mitochondrial permeability, and has shown promising results in cell cultures and rodent models of neurodegenerative conditions [5]. Based on these preliminary findings, a Phase III clinical trial was undertaken in Europe to evaluate the efficacy of olesoxime in ALS. Disappointingly, this study did not deliver therapeutic benefit on standard clinical endpoints such as survival, ALSFRS-r, manual muscle testing (MMT) or respiratory function [6]. A post-hoc analysis has now been performed using a state-of-the-art pharmaco-metabolomics approach with three specific goals: (1) to establish whether the combination of olesoxime and riluzole led to specific metabolic changes compared to riluzole therapy alone (2) to evaluate the relationship between metabolic patterns and clinical progression and (3) to evaluate if the prognostic value of early blood metabolomic profiles.

## Material and methods

### Patients

The TRO19622 (olesoxime) trial was a randomised, double-blind, placebo-controlled, phase III therapeutic trial which included 512 patients with ALS from 15 European centers between April 2009 and September 2011. Clinical and biological data were collected every three months for a total of 18 months. Data collection, data management and all study procedures were approved by local ethics committees in each participating country and written informed consent was obtained from all participant. A specific additional approval was obtained to perform metabolomic analyses on samples collected during the (NCT00868166) clinical trial from the local Ethical Committee (Comité de Protection des Personnes (CPP)-Ile de France VI- Groupe Hospitalier Pitié-Salpêtrière). Patients were diagnosed according to the El Escorial criteria [7] and those with a symptom duration of for more than six and less than 36 months were eligible. The demographic and clinical profile of study participants has been previously reported [6, 8]. Patients received 330 mg of olesoxime (treated group, Group O) or placebo (control group, Group P) once a day, in addition to riluzole for 18 months. We randomly selected 38 patients from Group O and 38 patients from Group P in order to determine their metabolomic profile at 1 month (V1) and 12 months (V12). Following rigorous quality assessments, 2 patients from the Group P have been excluded, and the final analysis included 38 patients from Group O and 36 participants from Group P.

### Clinical data

At V1 and V12, the following demographic and clinical data were documented: age at symptom onset, site of onset, disease duration from symptom onset, gender, the revised ALS Functional Rating Scale (ASLFRS-r) score, Slow Vital Capacity (SVC), Body Mass Index (BMI), muscle strength measured by manual muscle testing (MMT). If the patient had passed away by the V12 visit, this was also documented.

### Metabolomics experiments

A targeted, quantitative approach was implemented for the analysis of patient plasma samples. This method was based on the AbsolutIDQ™ p180 kit (Biocrates, Innsbruck, Austria) using a Flow Injection Analysis (FIA), and High-Performance Liquid Chromatography (HPLC-) Mass Spectrometry (MS/MS) assay. This assay kit enables the quantification of 188 metabolites. FIA is used for the semi-quantitative measurement of 146 hydrophobic molecules such as acylcarnitines, sphingomyelins (SM: type of sphingolipid consisting of phosphorylcholine and ceramide.) and phospholipids (lyso-, diacyl- and acyl- alkyl phosphatidylcholines (PC)). Forty-two polar metabolites (amino acids, hexoses and biogenic amines) were measured using HPLC. Specific metabolite ratios reflect metabolite-associated enzyme activity. This technique uses of isotope-labelled internal standards and provides quantitative results based on calibration curves and rigorous quality control analyses (QCs), as previously described [9]. Briefly, plasma are loaded onto a filter paper and dried in a stream of nitrogen for derivatisation with a solution of phenyl-isothiocyanate 5%. Subsequently, dried residues are extracted with methanol containing 5 mM ammonium acetate. The analysis is performed on a QTRAP® 5500 System (AB Sciex, Framingham, USA) with an FIA method or coupled to HPLC using a 5 μm Ascentis® Express C18 (4.6 × 250 mm) column. The MetIDQ® software (Biocrates) is used to calculate the concentrations of individual metabolites. The experiments are extensively validated using calibration curves and quality control protocols.

### Metabolomic modelling

Log-transformed metabolomics data were analysed by a multivariate approach using Simca P+ version 13.0 (Umetrics, Umeå, Sweden). First, Principal Component Analyses (PCA) were conducted, which is a descriptive multivariate analysis approach that efficiently identifies groups of samples based on their variable profiles (i.e. different metabolites). Grouping patterns, trends and outliers were examined on scatter plots. Orthogonal partial least-squares discriminant analyses (OPLS-DA or PLS-DA) were then performed. OPLS-DA identifies variations in peak areas between groups: variation in the measured data was partitioned into two blocks by the program, one containing variations that correlates with the class identifier and the other containing variations that are orthogonal to the first block and thus does not contribute to group discrimination. The OPLS-DA models were cross-validated by withholding one-seventh of the samples in seven simulations (each sample being omitted once) to avoid over-fitting. VIP values represent the importance of specific variables in OPLS -DA models, and the loadings characterize the relationship between the Y and X variables (lipids). We generated a loading plot that summarizes the most important variables in the separation; p(corr)[1] < 0 indicate variables associated with one group and p(corr)[1] > 0 represent variables associated with the second group. The overall quality of the models was appraised by the cumulative modeled variation in the X matrix R2X(cum), the cumulative modeled variation in the Y matrix R2Y(cum), and the cross validated predictive ability Q2(cum) values. Models were rejected if there was complete overlap of Q2 distributions (Q2(cum) < 0) or low classification rates (Q2(cum)< 0.05 and eigenvalues > 2). We considered a model robust if Q2> 40% and R2>50%, but these cut off values need to be confirmed under biological conditions. CV-ANOVA, ANalysis Of VAriance testing of Cross-Validated predictive residuals, is another diagnostic tool for assessing the reliability of the models. The set of multiple models resulting from the cross validation was used to calculate jack-knife uncertainty measures. We fixed the maximum number of iterations at 200 to ensure the convergence of the OPLS algorithm.

Based on these parameters, we optimized the models by excluding variables, so the most efficient model could be obtained from a minimal number of variables. Thus, we identified the most discriminant lipids based on the VIP and loading values scaled as correlation coefficients (pcorr).

We also used the biosigner algorithm for R (R project for statistical computing) [10] to identify the smallest pattern of variables from which a model can be generated with a significant regression coefficient. This algorithm entails sampling (bootstrap), VIP ranking and comprehensive performance evaluation by within-test-set permutations and half interval searches. The algorithm was independently wrapped around different machine-learning approaches, namely PLS-DA, Random Forest, and Support Vector Machines (SVM). The final training of the model is based on all samples from the dataset and the selected features. This stage involves the following steps: first, the dataset is split into training and testing datasets (by bootstrapping, controlling class proportion), then a model is built on the training set and prediction performance is evaluated on the test set. The features are thus ranked based on their contribution to the model. A feature is considered relevant if the random permutation of the intensities of other features in the test subsets does not alter significantly the accuracy. Finally, the dataset is restricted to the selected features and previous steps are repeated until the stability of selected features. The algorithm returns the tier of each feature for the different classifiers: 1) Tier S corresponds to the lipids which are significant in all selection steps; 2) Tier A is significant in all but the last selection, 3) Tier E regroups all previous rounds of selection. So, this analysis includes the same principles as performed by SIMCA® (PLS-DA) but overall, it is more robust as it is more restrictive. We have adjusted the ‘biosigner’ algorithm to modulate the size of the training and test sets, the number of bootstraps and to provide performance indicators in the independent test set: mean sensitivity, specificity, positive predictive value (PPV), and negative predictive value (NPV).

### Analysis strategy

**Fig 1:**
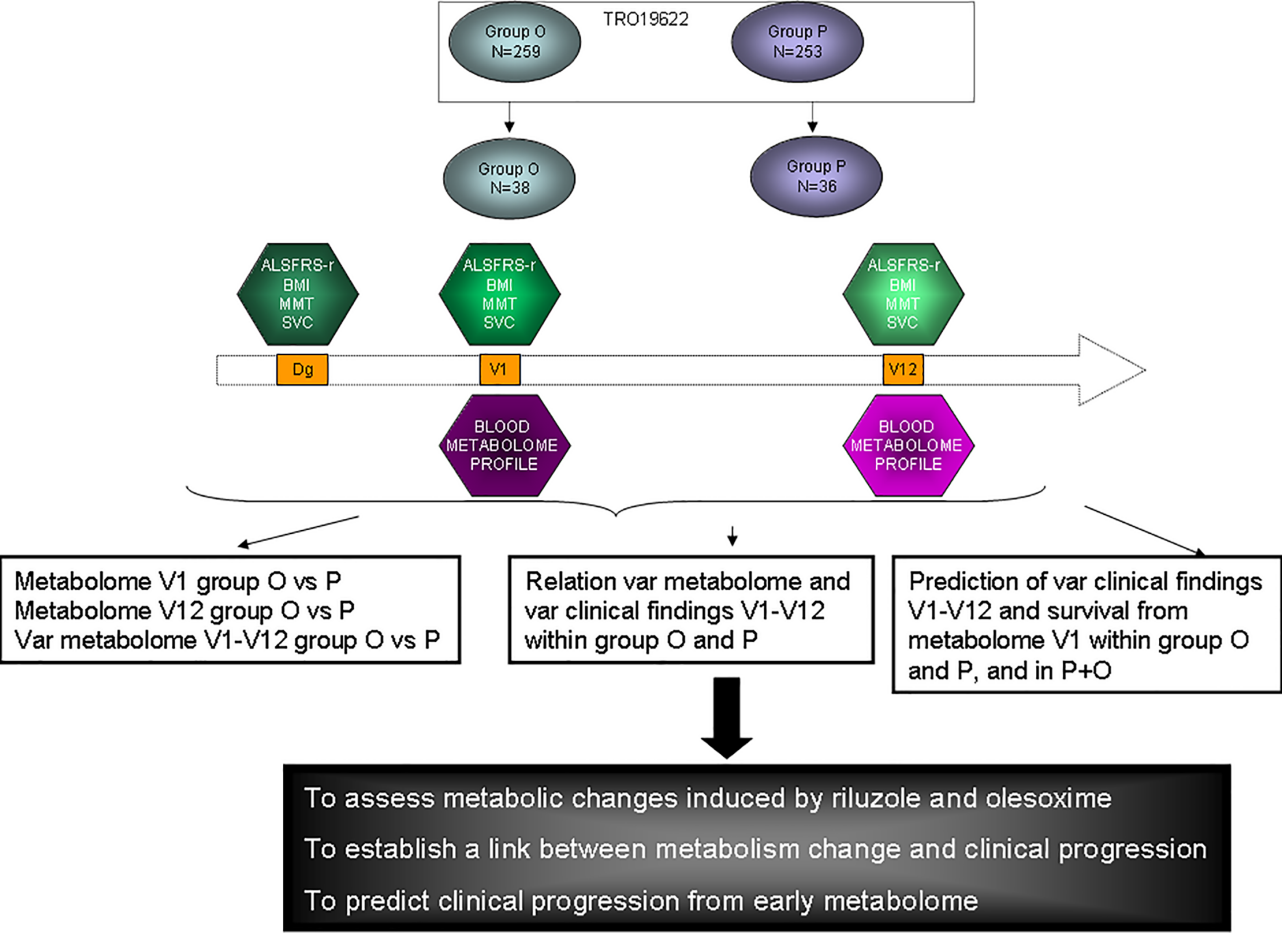
Global strategy of the analysis illustrating the times of sample collection, the parameters collected and the three main objectives of the study.

The methodology for data analysis is illustrated in Fig 1. First, the differences in metabolic profiles associated with treatment (group P vs. O) were described at 3 levels: V1, V12, and % of variation from V1 to V12. Second, we built multivariate models to evaluate the relationship between metabolomic variations between V1 and V12 and changes in clinical progression markers (ALSFRS-r, SVC, MMT, BMI modification) over the same period, for each group independently. Finally, we assessed the ability of the early metabolome profile at V1 to predict clinical progression defined by ALSFRS-r, SVC, MMT, BMI modification, in both treatment arms separately and together in order to identify metabolites indicative of disease progression. For each successful model, the Biosigner method was used to assess model performance over the entire cohort and to identity the most relevant metabolites. Subsequently, we have independently confirmed these findings on 1000 random training and test sets built from the cohorts (P or O or P+O), independently from the initial modelling (another algorithm on R and another person to perform such analysis). We reported the median performances of disease progression on the test set to specify the robustness of the modelling. Additionally, we built Venn diagrams based on the 15 most discriminating metabolites of the multivariate analyses to highlight the most promising candidate markers.

## Results

### Patient characteristics

The clinical and demographic profile of ALS patients in the two treatment groups are presented in Table 1. As the samples were randomly selected from the entire clinical trial cohort, no differences were observed between Group O and Group P, or between these groups and the general patient population included in the trial (Table 1) [6]. The complete database is made available as a supplementary file (S2 Table).

**Table 1:** Characteristics of patients at baseline (one month after the randomisation visit). Data from the entire cohort [6] are provided to confirm the representativeness of the population selected for the present study. Probability values correspond to the comparisons between Group O and Group P in this present study.

	Metabolomics study			Full study cohort	
	Group O(N = 38)	Group P(N = 36)	p	Olesoxime(N = 259)	Placebo(N = 253)
Gender (% men)	68.4%	69.4%	0.1	64.5%	64.8%
Age of onset	53.6 ± 11.5	50.2 ± 11.7	0.3	57.3 ± 11.2	55.7 ± 11.2
Site of onset			0.5		
Bulbar	13	19		51	50
Spinal	86	21		208	203
ALSFRS-r	39.5 ± 5.2	38.5 ± 6.1	0.5	39.1 ± 4.8	38.2 ± 5.3
BMI (kg/m^2^)	25.8 ± 3.2	24.3 ± 4.0	0.08	24.7 ± 3.4	24.8 ± 3.9
MMT	130.4 ± 15.9	126.5 ± 22.4	0.8	128/ ± 18	126 ± 18.8
SVC (%)	93.6 ± 13.9	94.4 ± 14.6	1.0	93.1 ± 14.6	93.1 ± 15.4
Diagnosis delay	9.3 ± 7.1	11.2 ± 4.8	0.9		

ALSFRS-r: Revised ALS Functional Rating Scale; BMI: Body Mass Index; MMT: Manual Muscle Testing; SVC: Slow Vital Capacity

#### Time-dependent metabolic modifications associated with olesoxime and/or riluzole

**Fig 2:**
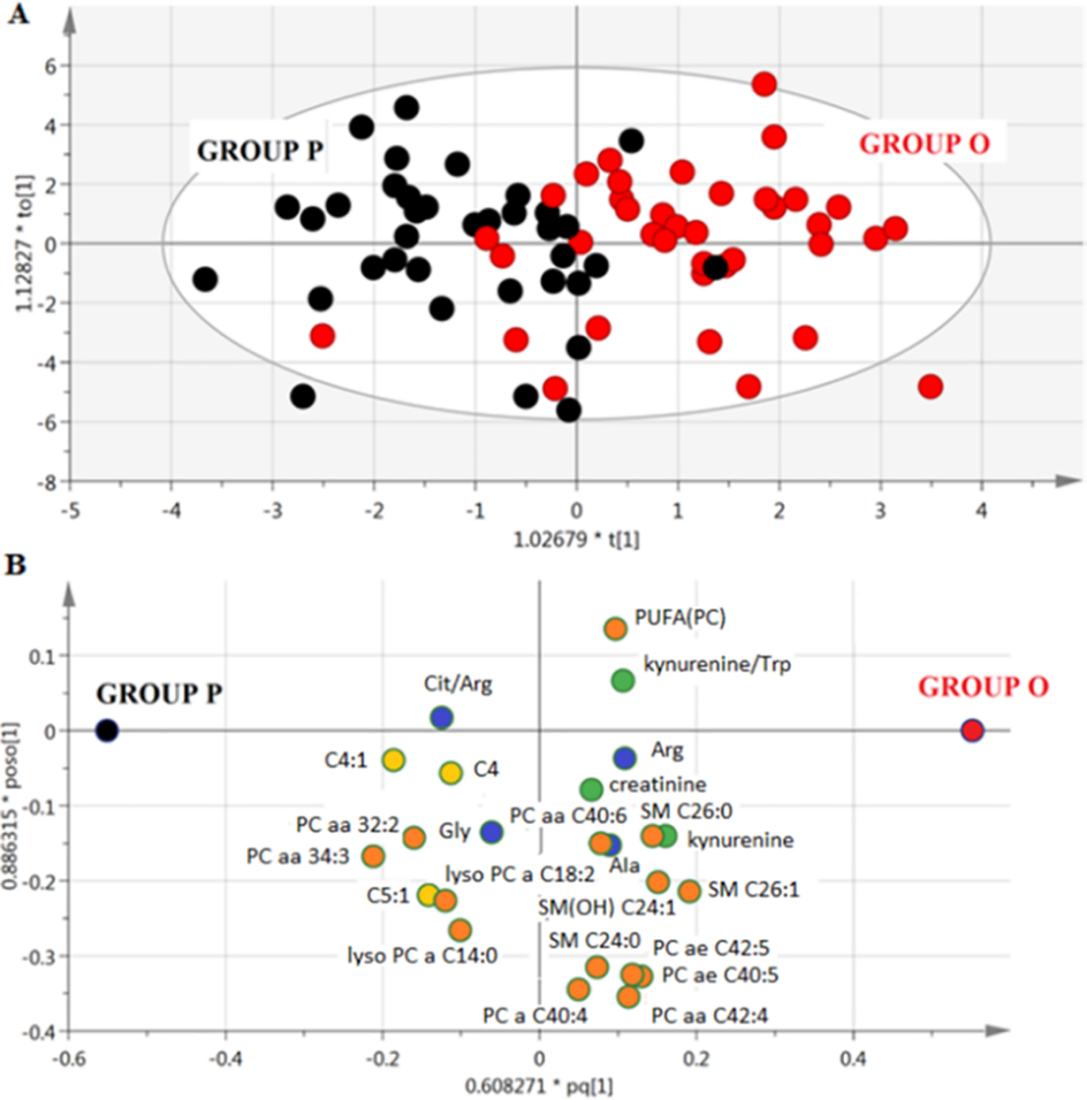
**Multivariate model from blood metabolome of ALS patients at V1**, A) Score scatter plot from OPLS-DA model discriminating patients in Group P (black) from patients in Group O (red), B) Loading scatter plot from OPLS-DA model showing the best discriminating metabolites. The position of the metabolites in the loading plot characterises the subjects represented in the score plot; variables near each other are positively correlated; variables opposite to each other are negatively correlated. Amino acids are represented in blue, complex lipids in orange, fatty acids in yellow and other molecules in green.

The OPLS-DA model derived from the metabolomic profile at V1 (p<5×10–6) allowed the two groups of patients (O and P) to be discriminated on the basis of 24 metabolites (Fig 2A and 2B, S1 Table). From the predictive variation between X (metabolites) and Y (blood samples) given by R2X(cum), the best models interpreted approximately 48% total variation in X. The predictability of treatment group membership from the biological data was acceptable (Q2Y(cum) = 0.35). Similarly, the OPLS-DA model effectively discriminated groups P and O on the basis of the 18 metabolites in the metabolome at V12 (p< 2×10–6) with a performance of R2X(cum) = 0.518 and Q2Y(cum) = 0.36.

**Fig 3:**
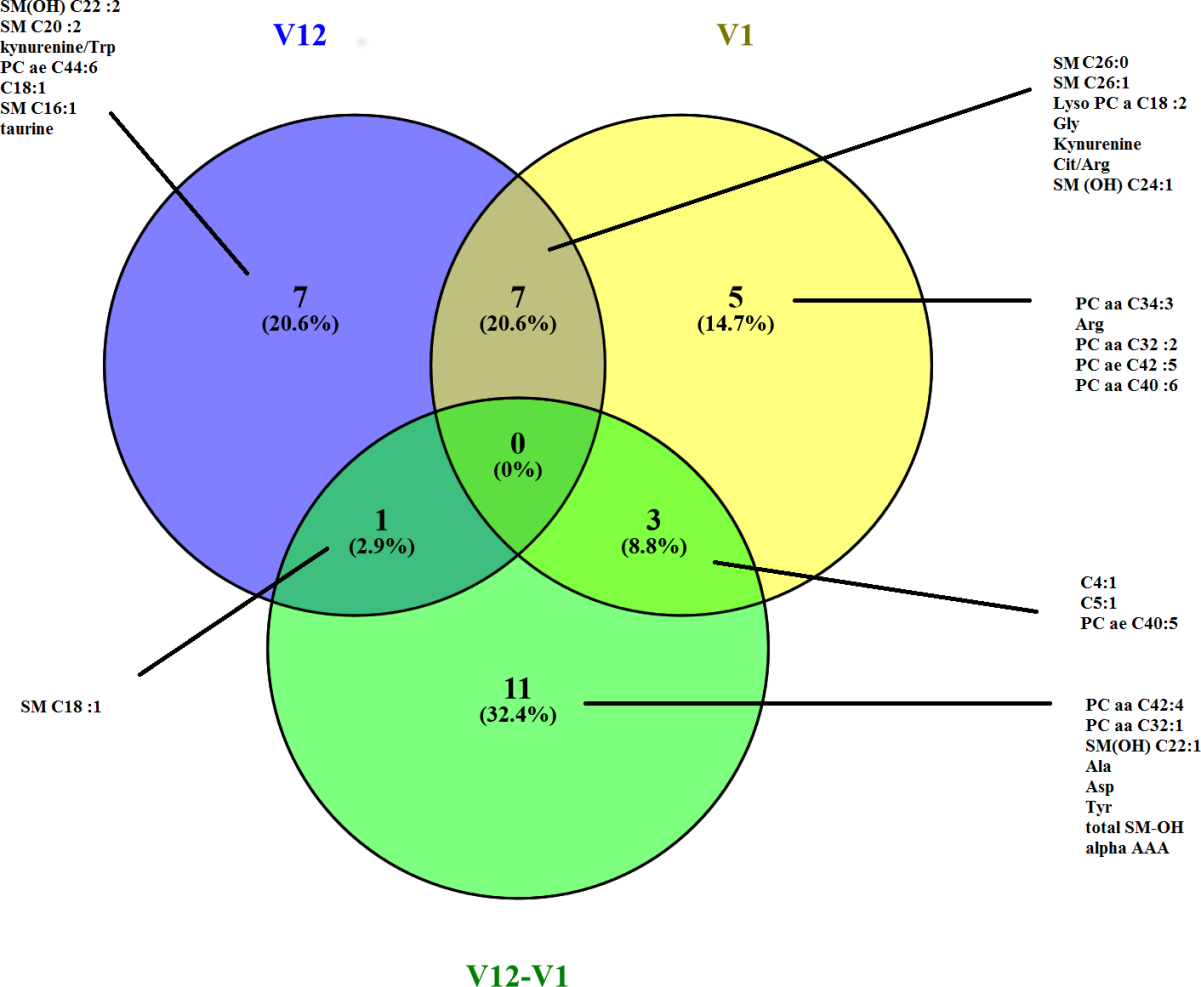
Venn diagram representing the 15 best discriminating metabolites between patients in Group P and patients in Group O at V1, V12 and over one year (V12-V1) in OPLS-DA models built from blood metabolomic profiles.

A third multivariate model elucidated the change in metabolite concentrations between V1 and V12 (p<4×10–6) based on 26 metabolites, with an R2X(cum) = 0.505 and Q2Y(cum) = 0.358. The Venn diagram highlights the metabolites that discriminate between treatment groups at V1, V12 and the change between V1 and V12 (Fig 3). The metabolites that best discriminated between Groups P and O were amino acids (Gly, citrulline/arginine), kynurenine and metabolites from lipid metabolism. Although these associations were significant for the profile of metabolites identified in the multivariate models, no significant association with treatment group was observed for the individual metabolites on their own based on univariate analysis.

### Association between metabolic changes and clinical progression

**Fig 4:**
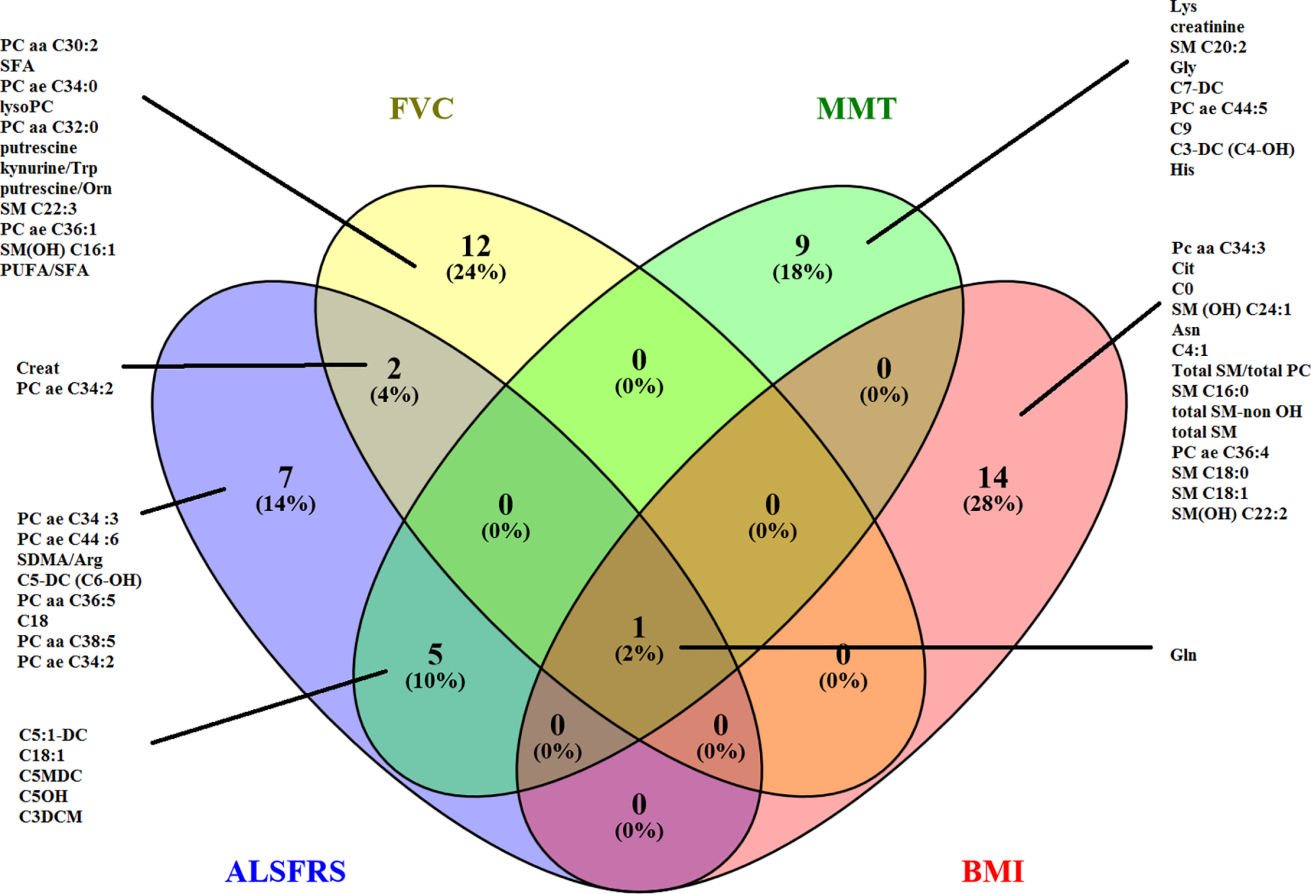
Venn diagram representing the 15 most discriminating metabolites in PLS-DA models which are associated with disease evolution (variation of ALSFRS-r, BMI, MMT, SVC over 1 year) in patients in Group P.

Multivariate models identified significant associations (not shown, p<0.01) between clinical changes in ALSFRS-r, SVC, MMT and BMI in Group P over time (V1 to V12) and alterations in metabolic profiles with satisfactory modelling quality. The Venn diagram (Fig 4) illustrates discriminating metabolites common to and distinct for the four disease progression variables. We observed that changes in glutamine were common to all variables. The most discriminating metabolites included creatine and metabolites related to lipid metabolism.

**Fig 5:**
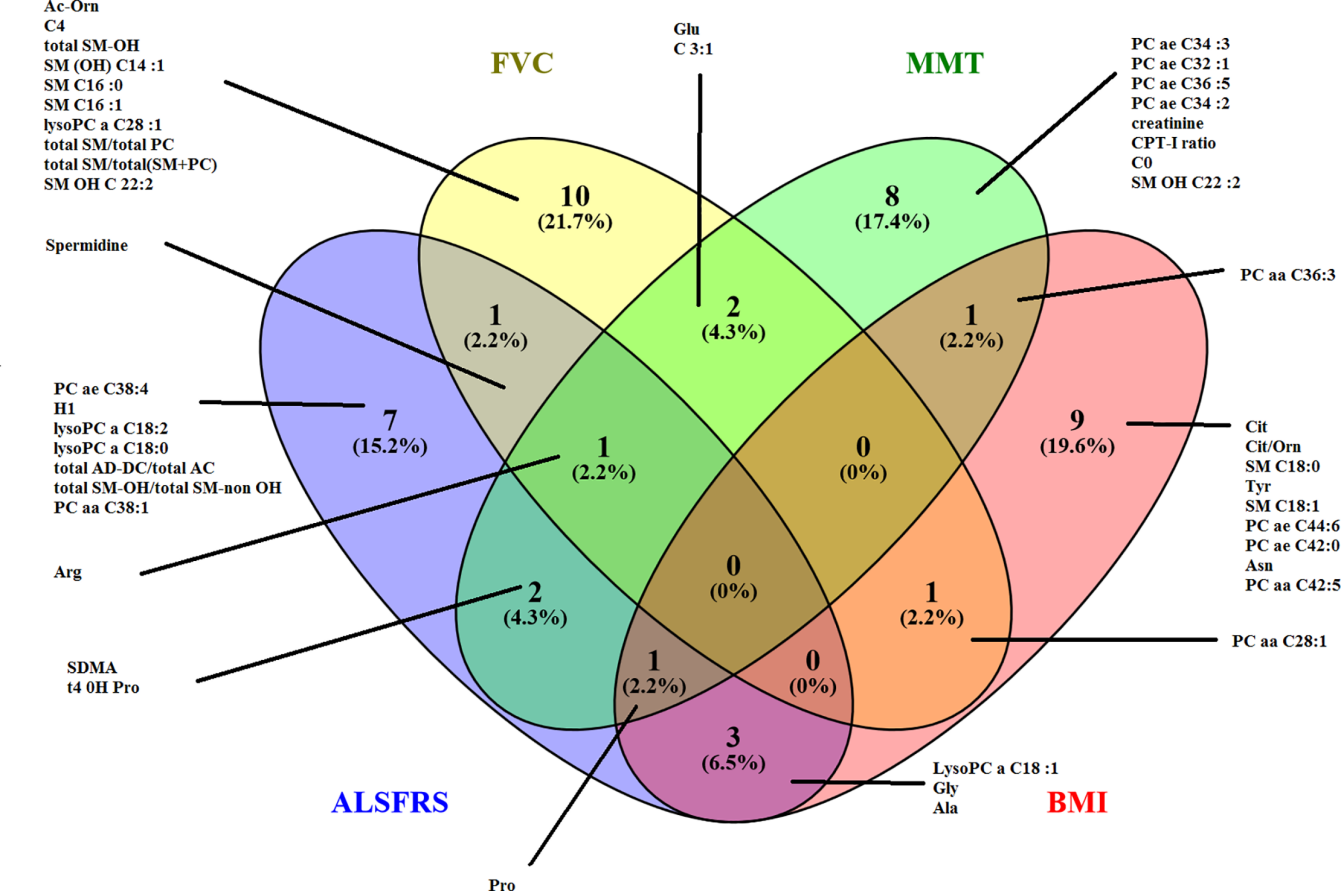
Venn diagram showing the 15 most discriminating metabolites in PLS-DA models which are associated with disease evolution (variation of ALSFRS-r, BMI, MMT, SVC over 1 year) in patients in Group O.

We applied the same strategy in Group O and obtained similarly acceptable PLS models with R2Xcum values between 0.38 and 0.61, R2Y(cum) between 0.62 and 0.913 and Q2Y(cum) from 0.33 to 0.712 (p<0.01). Numerous amino acids (arginine, proline, glycine, alanine, and glutamic acid), spermidine and some lipid metabolites demonstrated significant associations with progression markers in this patient group (Fig 5, S1 Table).

### Prediction of the clinical progression based on early metabolomic profiles

Similarly to the entire trial cohort, we did not observe any difference in change in ALSFRS-r (19.9 +-13.7 vs 22.3 +-17.0, p = 0.8), BMI (2.7+-6.7 vs 1.7 +-6.7 kg/m^2^, p = 0.4), MMT (20.8 +-18.0 vs 22.0 +-17.9, p = 0.6), SVFC (19.7 +-24.7 vs 21.1 +-26.6%, p = 0.7) between Groups O and P, reflecting the homogeneity in clinical progression. In Group P SIMCA® analysis revealed that, with the exception of MMT, it was possible to predict the variation of ALSFRS-r, BMI, and SVC from the metabolome at V1 with R2X(cum) between 0.28 and 0.56; R2Y(cum) between 0.31 and 0.46 and Q2(cum) between 0.23 and 0.3 (p-value <0.027). Metabolites that were associated with the evolution of clinical variables included taurine (ALSFRS-r, SVC), kynurenine/tryptophan (SVC, ALSFRS-r, MMT), C5:1, C4, putrescine/ornithine (ALSFRS-r, MMT) and SM C22:3 (ALSFRS-r, SVC). The independent modelling (RF) on 1000 training sets provided the following median performances in the test datasets: 100% sensitivity, 66.7% specificity, 66.7% positive predictive value, 100% negative predictive value. However, none of the models tested using this script was significant.

In Group O, SIMCA® analysis yielded satisfactory models to predict clinical progression with an R2X(cum) of 0.40 to 0.61; R2Y(cum) between 0.29 and 0.67 and Q2Y(cum) of 0.17 to 0.53 (p<0.04), except for BMI (p = 0.1). Most metabolites common to the different models were derived from lipids (SMC, SMOH, not shown) including PC aa C38:3 which is the most relevant findings as it was common to models explaining the variation of BMI, SVC, MMT. Biosigner algorithm resulted in no significant model but highlighted interesting metabolites: SM C24:1 (category S) was identified in the Random Forest model for SVC using the biosigner algorithm, and also in the PLS-DA model explaining changes in SVC, ALSFRS-r and MMT in the SIMCA® model. The independent modelling (RF) on 1000 training sets provided the following median performances on the test sets: 66.7% sensitivity, 66.7% specificity, 75% positive predictive value, 75% negative predictive value. When Groups O and P were pooled, the biosigner algorithm based on the 3 machine learning methods generated a significant model to explain the variation of SVC, with SM OH C22:2 (category S) as the most relevant marker, independently of the drug treatment. In this model, the sensitivity was 64.7%, the specificity 64.1%, the positive predictive value 31.1% and the negative predictive value 67.6%. In addition, SM C16:1(category A) was also discriminant for one out of 3 learning methods (i.e Random Forest). The independent modelling (RF) on 1000 training sets provided the following median performances on the test sets: 71.4% sensitivity, 71.4% specificity, 71.4% positive predictive value, 70.0% negative predictive value.

## Discussion

Our findings provide compelling proof of concept that pharmacometabolomic approaches add important insights in drug trials of ALS. We used standard statistical methods based on multivariate analyses (PLS, OPLS-DA), but also a novel algorithm to appraise models of prediction. The biosigner algorithm has been developed to provide a robust strategy for prediction, based primarily on bootstrapping and different learning methods, such as Random Forest, and Support Vector Machines [10]. Even if multivariate analysis based on SIMCA® is able to identify metabolites associated with clinical progression, its predictive power is weaker than that of the biosigner algorithm [10]. Beyond the context of therapeutic trials, the combination of rigorous phenotyping and advanced metabolomic approaches opens new perspectives to decipher molecular signatures underlying the clinical heterogeneity of ALS.

### Metabolic changes under treatment

The metabolic effect of riluzole has not been fully characterised in previous studies, which have overwhelmingly focused on glutamate effects [11], and the effect of olesoxime on TSPO (translocator protein) and VDAC (Voltage-Dependent Anion Channels) [4] has only been explored from a cholesterol metabolism perspective [8]. In this present study, glycine and the citrulline/arginine ratio were discriminating to a lesser extent in Group O than in Group P. Changes in glycine levels are consistent with the involvement of this amino acid in pathophysiology, including its role as a co-agonist of the N-methyl-D-aspartate receptor (NMDA-R) [12–14]. The modification of citrulline/arginine ratio may signal the involvement of mitochondrial alteration in ALS pathophysiology [15], including the inhibitory effect of NO on the respiratory chain [16], as well as its effect on oxidative stress [9, 17, 18]. The higher levels of kynurenine observed in Group O compared to Group P may be related to serotoninergic mechanisms and inflammation in ALS [19–21]. The kynurenine metabolic pathway includes neuroactive intermediates such as the NMDA-R agonist quinolinic acid and the NMDA-R antagonist picolinic acid [21]. As one of the neuroprotective actions of riluzole may include the inhibition of glutamate release and non-competitive post-synaptic inhibition of NMDA and AMPA receptors [22, 23], the increased kynurenine levels observed in Group O may be related to an attenuation of the effect of riluzole on these pathways due to a negative interaction with olesoxime treatment. Several lipids (sphingolipids, glycerophospholipids) were identified by the models discriminating the patients of two treatment groups. Despite previous reports that olesoxime does not affect cholesterol metabolism, its overall impact on lipid metabolism has never been explored [8]. Similarly, the effect of riluzole treatment on lipid metabolism has not been characterised, but it is conceivable that these alterations can be linked to changes in energy metabolism [17]. These findings merit further research, specifically focusing on the lipid alterations highlighted by this study.

### Relationship between metabolomic changes and clinical markers

In patients treated with riluzole alone, we identified creatinine as a key metabolite associated with clinical variables. The potential prognostic role of creatinine has been previously proposed in ALS [24, 25]. Glutamine, which is key blood amino acid and is closely linked with glutamate metabolism, was the most discriminating metabolite identified by each model of our analyses. Numerous studies underline the role of glutamate metabolism as central to ALS pathophysiology [11, 12] and our findings confirm the importance of this pathway. Glycine and Glutamate were identified by models exploring disease progression in Group O patients and these findings are consistent with previous reports showing that these alterations are time-dependent [9]. In addition, polyamines, which play biological roles in oxidative stress, NMDA receptor activation and autophagy, have been shown to be altered in patients with ALS and other neurodegenerative diseases [9].

### Disease prediction based on the early metabolomic profiles

In Group P, we found SM C22:3 (i.e sphingomyeline with 22 carbons and 3 unsaturations on fatty acids) as discriminating with respect to the prediction of several clinical indicators by overlapping statistical analyses, as was SM C34:1 in group O. Previous studies have implicated the role for sphingolipids in the pathogenesis in ALS; suggesting modulation of receptor-mediated signalling pathways, activity as lipid second messengers, perturbation of muscle function, and ceramide accumulation [26–28]. Nonetheless, further research is required to characterise the role of these metabolites in greater detail. As similar clinical progression was observed in Groups O and P, we investigated whether the early metabolomic profile can predict clinical progression irrespective of treatment group. Accordingly, we validated a model generated by the biosigner algorithm which predicted disease progression from the V1 metabolomics in patients from both groups. For all the models tested, two were relevant to predict SVC from SM C24:1 in group O and the other to predict SVC from SM OH C22:2 and SM C16:1 in group P+O. The performances of prediction obtained revealed correct models from one or two metabolites. We identified a sensitivity between 67% and 100%, a specificity between 66.7 and 71.4%, a positive predictive value between 66 and 75% and a negative predictive value between 70% and 100% in the test sets, which is noteworthy in a highly heterogeneous disease. A signal was again observed for a sphingolipid. In a follow-up study, we will specifically investigate these pathways in more detail by a dedicated lipidomics approach [29].

A limitation of the study is the lack of external validation, however our strategy was designed to include robust internal validation with bootstraps.

In conclusion, we have demonstrated an innovative strategy for detailed post-hoc analysis of metabolomic profiles in a clinical trial of ALS. We showed that treatment with olesoxime and riluzole modifies different metabolic pathways. The metabolic insights provided by these analyses regarding the specific mechanism of action of these drugs have implications for the development of other drugs in ALS and other neurodegenerative diseases. The prediction of disease progression from early metabolomic profiles is a particularly promising avenue. Current clinical trials typically include systematic sampling of biomarkers therefore pharmaco-metabolomics is an exciting novel approach to capitalise from this material. Further studies are required to develop these approaches to meaningful and efficient drug development strategies.

## Supporting information

S1 Table List of metabolites analysed and notification of metabolites involved in the multivariate models explaining the treatment (O vs P) and disease progression (in the overall population) from V1 metabolome (i.e. corresponding to the Figs 2 and 4, respectively). (DOC)

S2 Table Database of the study. (XLS)
