# Supplementary material for: A pharmaco-metabolomics approach in a clinical trial of ALS: Identification of predictive markers of progression
Source: PLoS One. 2018 Jun 5;13(6):e0198116. doi: 10.1371/journal.pone.0198116 (PMC5988280; doi:10.1371/journal.pone.0198116)
Supplement: S1 Table — (DOC) [file pone.0198116.s001.doc]

| **Metabolite** | **Analytical platform** | **HMDB ID** | **V1**  **O vs P** | **Survival**  **O + P** |
| --- | --- | --- | --- | --- |
| **C0** | FIA | HMDB00062 |  | X |
| **C10** | FIA | HMDB00651 |  |  |
| **C10:1** | FIA | HMDB13205 |  |  |
| **C10:2** | FIA | HMDB13325 |  | X |
| **C12** | FIA | HMDB02250 |  |  |
| **C12:1** | FIA | HMDB13326 |  |  |
| **C12-DC** | FIA |  |  |  |
| **C14** | FIA | HMDB05066 |  |  |
| **C14:1** | FIA | HMDB02014 HMDB13329 |  |  |
| **C14:1-OH** | FIA |  |  |  |
| **C14:2** | FIA | HMDB13331 |  |  |
| **C14:2-OH** | FIA |  |  |  |
| **C16** | FIA | HMDB00222 |  |  |
| **C16:1** | FIA | HMDB13207 |  |  |
| **C16:1-OH** | FIA | HMDB13333 |  |  |
| **C16:2** | FIA |  |  |  |
| **C16:2-OH** | FIA |  |  |  |
| **C16-OH** | FIA | HMDB13337 HMDB13336 |  |  |
| **C18** | FIA | HMDB00848 |  |  |
| **C18:1** | FIA | HMDB05065 |  |  |
| **C18:1-OH** | FIA | HMDB13340 HMDB13339 |  |  |
| **C18:2** | FIA | HMDB06469 |  |  |
| **C2** | FIA | HMDB00201 |  |  |
| **C3** | FIA | HMDB00824 |  |  |
| **C3:1** | FIA |  |  |  |
| **C3-DC (C4-OH)** | FIA |  |  |  |
| **C3-OH** | FIA |  |  |  |
| **C4** | FIA | HMDB02013 | X |  |
| **C4:1** | FIA |  | X |  |
| **C5** | FIA | HMDB00688 |  |  |
| **C5:1** | FIA | HMDB02366 | X |  |
| **C5:1-DC** | FIA |  |  |  |
| **C5-DC (C6-OH)** | FIA |  |  |  |
| **C5-M-DC** | FIA |  |  |  |
| **C5-OH (C3-DC-M)** | FIA |  |  |  |
| **C6 (C4:1-DC)** | FIA |  |  |  |
| **C6:1** | FIA |  |  |  |
| **C7-DC** | FIA |  |  |  |
| **C8** | FIA | HMDB00791 |  |  |
| **C9** | FIA |  |  |  |
| **lysoPC a C14:0** | FIA | HMDB10379 | X |  |
| **lysoPC a C16:0** | FIA | HMDB10382 |  |  |
| **lysoPC a C16:1** | FIA | HMDB10383 |  |  |
| **lysoPC a C17:0** | FIA | HMDB12108 |  | X |
| **lysoPC a C18:0** | FIA | HMDB10384 |  |  |
| **lysoPC a C18:1** | FIA | HMDB02815 |  |  |
| **lysoPC a C18:2** | FIA | HMDB10386 | X |  |
| **lysoPC a C20:3** | FIA | HMDB10394 |  |  |
| **lysoPC a C20:4** | FIA | HMDB10395 |  |  |
| **lysoPC a C24:0** | FIA | HMDB10405 |  |  |
| **lysoPC a C26:0** | FIA |  |  |  |
| **lysoPC a C26:1** | FIA |  |  |  |
| **lysoPC a C28:0** | FIA |  |  |  |
| **lysoPC a C28:1** | FIA |  |  |  |
| **Total lysoPC/total PC** |  |  |  | X |
| **Total SM/total PC** |  |  |  | X |
| **Total SM/total (SM+PC)** |  |  |  | X |
| **PC aa C24:0** | FIA |  |  |  |
| **PC aa C26:0** | FIA |  |  |  |
| **PC aa C28:1** | FIA | HMDB07899 |  | X |
| **PC aa C30:0** | FIA | HMDB07869 |  |  |
| **PC aa C30:2** | FIA | HMDB07903 |  |  |
| **PC aa C32:0** | FIA | HMDB07871 |  |  |
| **PC aa C32:1** | FIA | HMDB07969 |  |  |
| **PC aa C32:2** | FIA | HMDB07874 | X |  |
| **PC aa C32:3** | FIA | HMDB07876 |  |  |
| **PC aa C34:1** | FIA | HMDB07972 |  |  |
| **PC aa C34:2** | FIA | HMDB07973 |  |  |
| **PC aa C34:3** | FIA | HMDB07974 | X |  |
| **PC aa C34:4** | FIA | HMDB07883 |  |  |
| **PC aa C36:0** | FIA | HMDB08036 |  |  |
| **PC aa C36:1** | FIA | HMDB08037 |  |  |
| **PC aa C36:2** | FIA | HMDB00593 |  |  |
| **PC aa C36:3** | FIA | HMDB07980 |  |  |
| **PC aa C36:4** | FIA | HMDB08138 |  | X |
| **PC aa C36:5** | FIA | HMDB07984 |  |  |
| **PC aa C36:6** | FIA | HMDB07892 |  |  |
| **PC aa C38:0** | FIA | HMDB07893 |  |  |
| **PC aa C38:1** | FIA | HMDB07894 |  |  |
| **PC aa C38:3** | FIA | HMDB08046 |  | X |
| **PC aa C38:4** | FIA | HMDB08048 |  | X |
| **PC aa C38:5** | FIA | HMDB07989 |  |  |
| **PC aa C38:6** | FIA | HMDB07991 |  | X |
| **PC aa C40:1** | FIA | HMDB07993 |  |  |
| **PC aa C40:2** | FIA | HMDB08276 |  |  |
| **PC aa C40:3** | FIA | HMDB08119 |  |  |
| **PC aa C40:4** | FIA | HMDB08054 | X | X |
| **PC aa C40:5** | FIA |  |  | X |
| **PC aa C40:6** | FIA | HMDB08057 | X | X |
| **PC aa C42:0** | FIA | HMDB08058 |  | X |
| **PC aa C42:1** | FIA | HMDB08059 |  | X |
| **PC aa C42:2** | FIA | HMDB08284 |  |  |
| **PC aa C42:4** | FIA | HMDB08285 | X |  |
| **PC aa C42:5** | FIA | HMDB08287 |  |  |
| **PC aa C42:6** | FIA | HMDB08288 |  |  |
| **PC ae C30:0** | FIA | HMDB13341 |  |  |
| **PC ae C30:1** | FIA | HMDB13402 |  |  |
| **PC ae C30:2** | FIA | HMDB13410 |  |  |
| **PC ae C32:1** | FIA | HMDB13404 |  |  |
| **PC ae C32:2** | FIA | HMDB13411 |  |  |
| **PC ae C34:0** | FIA | HMDB13405 |  |  |
| **PC ae C34:1** | FIA | HMDB13426 |  |  |
| **PC ae C34:2** | FIA | HMDB11151 |  |  |
| **PC ae C34:3** | FIA | HMDB13413 |  |  |
| **PC ae C36:0** | FIA | HMDB13406 |  |  |
| **PC ae C36:1** | FIA | HMDB13414 |  |  |
| **PC ae C36:2** | FIA | HMDB13418 |  |  |
| **PC ae C36:3** | FIA | HMDB13429 |  |  |
| **PC ae C36:4** | FIA | HMDB13407 |  |  |
| **PC ae C36:5** | FIA | HMDB13415 |  |  |
| **PC ae C38:0** | FIA | HMDB13408 |  |  |
| **PC ae C38:1** | FIA | HMDB13416 |  |  |
| **PC ae C38:2** | FIA | HMDB13431 |  |  |
| **PC ae C38:3** | FIA | HMDB13439 |  |  |
| **PC ae C38:4** | FIA | HMDB13420 |  |  |
| **PC ae C38:5** | FIA | HMDB13432 |  |  |
| **PC ae C38:6** | FIA | HMDB13409 |  |  |
| **PC ae C40:1** | FIA | HMDB13433 |  | X |
| **PC ae C40:2** | FIA | HMDB13437 |  |  |
| **PC ae C40:3** | FIA | HMDB13445 |  |  |
| **PC ae C40:4** | FIA | HMDB13442 |  |  |
| **PC ae C40:5** | FIA | HMDB13444 | X |  |
| **PC ae C40:6** | FIA | HMDB13422 |  |  |
| **PC ae C42:0** | FIA | HMDB13443 |  |  |
| **PC ae C42:1** | FIA | HMDB13434 |  |  |
| **PC ae C42:2** | FIA | HMDB13438 |  |  |
| **PC ae C42:3** | FIA | HMDB13458 |  |  |
| **PC ae C42:4** | FIA | HMDB13448 |  |  |
| **PC ae C42:5** | FIA | HMDB13451 | X |  |
| **PC ae C44:3** | FIA | HMDB13449 |  |  |
| **PC ae C44:4** | FIA | HMDB13453 |  |  |
| **PC ae C44:5** | FIA | HMDB13456 |  |  |
| **PC ae C44:6** | FIA | HMDB13450 |  |  |
| **PUFA** |  |  | X |  |
| **SM (OH) C14:1** | FIA |  |  |  |
| **SM (OH) C16:1** | FIA |  |  |  |
| **SM (OH) C22:1** | FIA |  |  |  |
| **SM (OH) C22:2** | FIA |  |  |  |
| **SM (OH) C24:1** | FIA |  | X |  |
| **SM C16:0** | FIA |  |  |  |
| **SM C16:1** | FIA |  |  |  |
| **SM C18:0** | FIA |  |  |  |
| **SM C18:1** | FIA |  |  |  |
| **SM C20:2** | FIA |  |  |  |
| **SM C22:3** | FIA |  |  |  |
| **SM C24:0** | FIA |  | X |  |
| **SM C24:1** | FIA |  |  |  |
| **SM C26:0** | FIA |  | X |  |
| **SM C26:1** | FIA |  | X |  |
| **3-Nitrotyrosine** | HPLC | HMDB01904 |  |  |
| **4-Hydroxyproline** | HPLC | HMDB00725 |  |  |
| **ADMA (asymetric dimethylarginine)** | HPLC | HMDB01539 |  |  |
| **Carnosine** | HPLC | HMDB00033 |  |  |
| **Alpha-AAA (alpha aminoadipic acid)** | HPLC |  |  |  |
| **Creatinine** | HPLC | HMDB00562 | X |  |
| **Dopamine** | HPLC | HMDB00073 |  |  |
| **Glycine** | HPLC | HMDB00123 | X |  |
| **Histamine** | HPLC | HMDB00870 |  |  |
| **L-Alanine** | HPLC | HMDB00161 | X |  |
| **L-Arginine** | HPLC | HMDB00517 | X |  |
| **L-Asparagine** | HPLC | HMDB00168 |  | X |
| **L-Aspartic acid** | HPLC | HMDB00191 |  |  |
| **L-Citrulline** | HPLC | HMDB00904 | X |  |
| **L-DOPA** | HPLC | HMDB00181 |  |  |
| **L-Glutamic acid** | HPLC | HMDB00148 |  | X |
| **L-Glutamine** | HPLC | HMDB00641 |  | X |
| **L-Histidine** | HPLC | HMDB00177 |  |  |
| **L-Isoleucine** | HPLC | HMDB00172 |  |  |
| **L-Kynurenine** | HPLC | HMDB00684 | X | X |
| **L-Leucine** | HPLC | HMDB00687 |  |  |
| **L-Lysine** | HPLC | HMDB00182 |  |  |
| **L-Methionine** | HPLC | HMDB00696 |  | X |
| **L-Ornithine** | HPLC | HMDB00214 |  | X |
| **L-Phenylalanine** | HPLC | HMDB00159 |  |  |
| **L-Proline** | HPLC | HMDB00162 |  |  |
| **L-Serine** | HPLC | HMDB00187 |  |  |
| **L-Threonine** | HPLC | HMDB00167 |  |  |
| **L-Tryptophan** | HPLC | HMDB00929 | X |  |
| **L-Tyrosine** | HPLC | HMDB00158 |  |  |
| **L-Valine** | HPLC | HMDB00883 |  |  |
| **Methionin Sulfoxide** | HPLC |  |  |  |
| **N-Acetylornithine** | HPLC | HMDB03357 |  |  |
| **Phenylethylamine** | HPLC | HMDB12275 |  |  |
| **Putrescine** | HPLC | HMDB01414 |  | X |
| **SDMA** | HPLC | HMDB03334 |  |  |
| **Serotonin** | HPLC | HMDB00259 |  |  |
| **Spermidine** | HPLC | HMDB01257 |  |  |
| **Spermine** | HPLC | HMDB01256 |  |  |
| **Taurine** | HPLC | HMDB00251 |  |  |
| **t4-OH-Pro** | HPLC |  |  |  |
| **Total dimethylarginine** | HPLC |  |  |  |
| **H1** | HPLC |  |  |  |
